# The Functional Neuroanatomy of Lexical Tone Perception: An Activation Likelihood Estimation Meta-Analysis

**DOI:** 10.3389/fnins.2018.00495

**Published:** 2018-07-24

**Authors:** Baishen Liang, Yi Du

**Affiliations:** ^1^CAS Key Laboratory of Behavioral Science, CAS Center for Excellence in Brain Science and Intelligence Technology, Institute of Psychology, Chinese Academy of Sciences, Beijing, China; ^2^Department of Psychology, University of Chinese Academy of Sciences, Beijing, China

**Keywords:** lexical tone, phoneme, prosody, speech perception, meta-analysis, neuroimaging

## Abstract

In tonal language such as Chinese, lexical tone serves as a phonemic feature in determining word meaning. Meanwhile, it is close to prosody in terms of suprasegmental pitch variations and larynx-based articulation. The important yet mixed nature of lexical tone has evoked considerable studies, but no consensus has been reached on its functional neuroanatomy. This meta-analysis aimed at uncovering the neural network of lexical tone perception in comparison with that of phoneme and prosody in a unified framework. Independent Activation Likelihood Estimation meta-analyses were conducted for different linguistic elements: lexical tone by native tonal language speakers, lexical tone by non-tonal language speakers, phoneme, word-level prosody, and sentence-level prosody. Results showed that lexical tone and prosody studies demonstrated more extensive activations in the right than the left auditory cortex, whereas the opposite pattern was found for phoneme studies. Only tonal language speakers consistently recruited the left anterior superior temporal gyrus (STG) for processing lexical tone, an area implicated in phoneme processing and word-form recognition. Moreover, an anterior-lateral to posterior-medial gradient of activation as a function of element timescale was revealed in the right STG, in which the activation for lexical tone lied between that for phoneme and that for prosody. Another topological pattern was shown on the left precentral gyrus (preCG), with the activation for lexical tone overlapped with that for prosody but ventral to that for phoneme. These findings provide evidence that the neural network for lexical tone perception is hybrid with those for phoneme and prosody. That is, resembling prosody, lexical tone perception, regardless of language experience, involved right auditory cortex, with activation localized between sites engaged by phonemic and prosodic processing, suggesting a hierarchical organization of representations in the right auditory cortex. For tonal language speakers, lexical tone additionally engaged the left STG lexical mapping network, consistent with the phonemic representation. Similarly, when processing lexical tone, only tonal language speakers engaged the left preCG site implicated in prosody perception, consistent with tonal language speakers having stronger articulatory representations for lexical tone in the laryngeal sensorimotor network. A dynamic dual-stream model for lexical tone perception was proposed and discussed.

## Introduction

During spoken language comprehension, various speech elements (phoneme, lexical tone, and prosody) interplay simultaneously to convey linguistic and paralinguistic information. Phoneme (namely segmental phoneme including consonant and vowel), which is the smallest contrastive unit of speech that distinguishes different words, changes rapidly in formants via distinct gestures of articulators (e.g., lips and tongue). Prosody, the determinant for stress and intonation (linguistic prosody) or a supplementary expression of emotions (affective prosody), varies in pitch at the suprasegmental length of a syllable, a phrase or a sentence as a result of laryngeal vibration. In tonal languages, lexical tone is usually recognized as a suprasegmental form of phoneme and called as tone phoneme or “toneme” (Chao, 1968). As shown in Table 1, which gives a summary of different speech elements from perspectives of acoustic-phonetic feature, place of articulation and linguistic function, lexical tone incorporates properties of both phoneme and prosody. On the one side, tone functions as phoneme to account for lexical meaning; on the other side, it changes in the level and contour of pitch across one syllable and is shaped by movements of larynx, which is analogous to prosody.

**Table 1:** A summary of different speech elements.

**Element**		**Length**	**Acoustic-phonetic feature**	**Place of articulation**	**Linguistic function**
Lexical tone (tone phoneme)		Suprasegmental	Level and contour of the fundamental frequency	Larynx	Determine lexical meaning
Segmental Phoneme	Consonant	Segmental	Voice onset time and formant transitions	Tongue, lips, teeth, palate	Determine lexical meaning
	Vowel	Segmental	Regions of the 1st and 2nd formants	Tongue, lips	Determine lexical meaning
Prosody	Word prosody	Suprasegmental	Level and contour of the fundamental frequency	Larynx	Pragmatic (intonation, stress, rhythm, emotion)
	Sentence prosody	Suprasegmental	Level and contour of the fundamental frequency	Larynx	Pragmatic (intonation, stress, rhythm, emotion)

The unique properties of lexical tone have triggered wide research interest in its neural substrates, which, however, are still controversial. One of the debates lies in hemispherical asymmetry. Using various methodologies, studies have reported either right (Ren et al., 2009; Ge et al., 2015) or left (Xi et al., 2010; Gu et al., 2013) biased activation for lexical tone perception. The discrepancy could be partially reconciled by the modulatory effect of language experience in the interplay of bottom-up and top-down processes during lexical tone perception (Zatorre and Gandour, 2008). Moreover, as speech comprehension incorporates multiple perceptual and cognitive mechanisms (Hickok and Poeppel, 2007), including spectrotemporal analysis in bilateral STG of the ventral auditory stream (Hullett et al., 2016) and sensorimotor integration by the left-lateralized articulatory network in the dorsal auditory stream (Du et al., 2014, 2016), the perception of lexical tone may dynamically recruit distinct asymmetric processes.

Several models on speech perception have offered insights into the hemispherical asymmetry of lexical tone perception in auditory cortices. According to the model of spectrotemporal resolution, the spectral and temporal acoustical properties of signals could predict the relative specialization of the right and left auditory cortices (Zatorre et al., 2002). Whereas, the Asymmetric Sampling in Time (AST) model (Poeppel, 2003) has suggested a preferential tuning of the left and right superior temporal cortices in processing auditory information in short (20–50 ms, ~4 Hz) and long (150–250 ms, ~40 Hz) temporal integration window, respectively. Indeed, previous studies have supported a left and right biased neural foundation for the perception of phoneme and prosody, separately (DeWitt and Rauschecker, 2012; Witteman et al., 2012; Belyk and Brown, 2013). Hence, given its suprasegmental pitch variations, which is similar to prosody, right asymmetric activations in auditory cortices for lexical tone perception were predicted.

Moreover, human auditory cortices have demonstrated local gradients as a function of spectrotemporal modulation rate preference. Using functional magnetic resonance imaging (fMRI, Santoro et al., 2014) and electrocorticography (ECoG, Hullett et al., 2016), recent studies have found peak tuning for high spectral modulation rates near the anterior-lateral aspect of Heschl's gyrus and preference for low temporal modulation rates along the lateral aspect of planum temporale. Meanwhile, anterior-posterior hierarchical representations of speech stimuli with decreasing timescale (phrase-syllable-phoneme, DeWitt and Rauschecker, 2012) and increasing timescale (word-sentence-paragraph, Lerner et al., 2011) have both been reported on bilateral STG. Thus, considering requirements on spectrotemporal modulation rate tuning and unit timescales, we hypothesized that perception of lexical tone might activate an STG subregion that lies between activation of phoneme and activation of prosody.

In addition, sensorimotor integration has been proposed to compensate for speech perception (Hickok and Poeppel, 2007; Rauschecker and Scott, 2009). This account posits an internal model generated by the listener's speech motor system, e.g., Broca's area and left motor/premotor cortex, to anticipate sensory sequences of the speaker's articulatory gestures. Such predictions may impose phonological constraints to auditory representations in sensorimotor interface areas, including the left posterior STG (pSTG) and inferior parietal lobule (IPL). Sensorimotor integration has been suggested to facilitate speech perception, especially in degraded listening environments (Du et al., 2014) and aging populations (Du et al., 2016). Indeed, it is shown that the left and right motor networks predominately support the perception of phoneme (Du et al., 2014) and prosody (Sammler et al., 2015), respectively, while bilateral articulatory regions were activated in lexical tone perception (Si et al., 2017). Furthermore, as different linguistic elements are pronounced by various places of articulation (e.g., lips and tongue for phoneme vs. larynx for prosody and lexical tone), distinct areas along the motor and premotor cortices might be involved according to the somatomotor topography (Schomers and Pulvermüller, 2016). Although many neuroimaging studies have investigated the recruitment of motor areas in speech perception, sparse meta-analyses and reviews have highlighted this motor function (Skipper et al., 2017). Hence, the property of sensorimotor integration of lexical tone perception in terms of hemispherical asymmetry and local topography in comparison with that of phoneme and prosody is unclear. We predicted that perception of lexical tone might engage bilateral speech motor areas with local motor activation co-located with that for prosody.

Meta-analysis of previous published fMRI and positron emission tomography (PET) studies reveals robust convergence of activation patterns immune from experimental bias, and is predominant in comparing neural networks across different tasks and stimuli. Using ALE algorithm (Eickhoff et al., 2009, 2012), a recent meta-analysis on lexical tone has demonstrated convergent activations in bilateral inferior prefrontal and superior temporal regions as well as the right caudate during lexical tone processing using both perception and production tasks (Kwok et al., 2017). Differently, the current meta-analysis focused on lexical tone perception only and compared the neuroanatomy of lexical tone perception with that of phoneme perception and prosody perception. In particular, this study aimed at providing a clearer panorama for neural underpinnings of perceiving different linguistic elements, from the aspects of hemispherical asymmetry and topographic representations in the ventral and dorsal auditory streams.

## Materials and methods

### Search strategy

Papers for analyses on three types of speech elements (phoneme, lexical tone, and prosody) were searched in PubMed database (www.pubmed.com) independently. Titles or abstracts of studies must contain the following keywords: “tone” (or “tonal” and “tones”) and “lexical” (or “Mandarin,” “Chinese,” “Cantonese,” and “Thai”) for lexical tone; “phoneme” (or “consonant,” “vowel,” and “segment”) for phoneme; and “prosody” or “intonation” for prosody, crossed with “fMRI,” “functional magnetic resonance imaging,” “BOLD,” “PET,” and “positron emission tomography.” All studies included were published in peer-reviewed journals written in English as of October 2017. Relevant studies from references of previous meta-analyses (Belyk and Brown, 2013; Kwok et al., 2017) not identified in this process were manually selected and screened.

### Screening process

**Figure 1:**
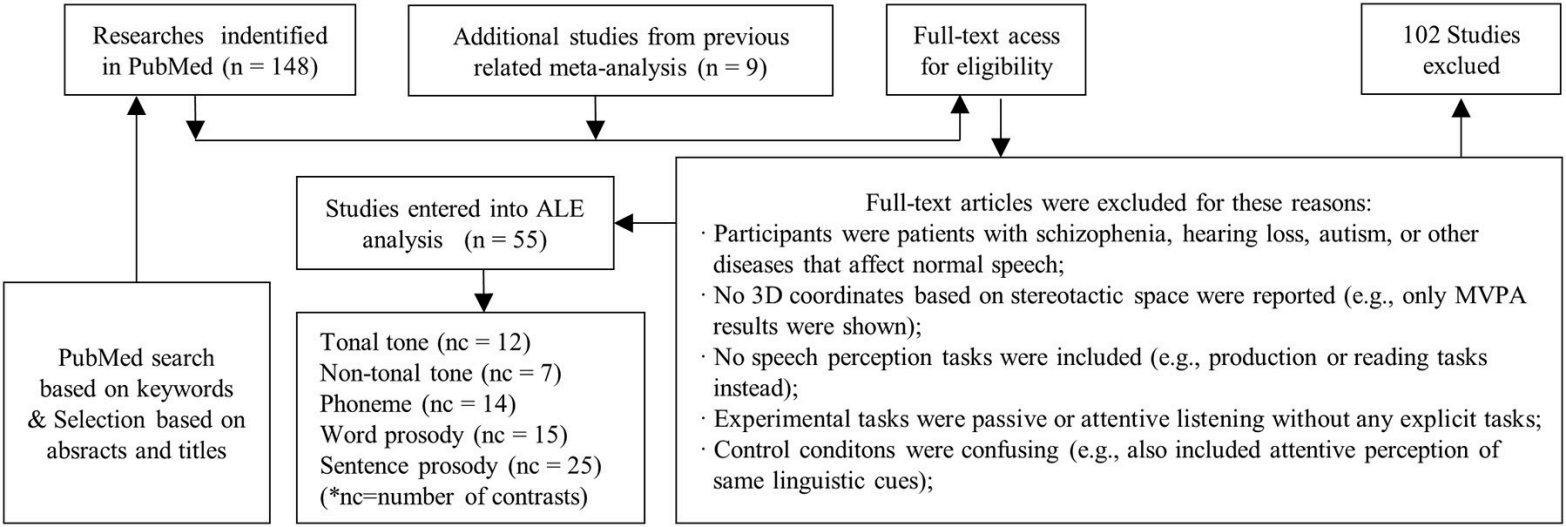
Procedure of selection. Papers selected from PubMed and previous meta-analysis were screened manually following the criteria. Contrasts from selected papers were grouped into five categories, which were then entered into meta-analysis independently. Note that, the number of studies entered into analysis was smaller than the sum of contrasts in each condition, because one selected study may contain more than one contrast.

Studies were screened in full-text against the criteria of eligibility outlined in Figure 1, which depicts the process of screening.

Studies were included if they met the following criteria: (1) participants were young healthy adults without any hearing, psychiatric or neurological disorders, or brain abnormalities; (2) whole brain analysis from fMRI or PET on 3D coordinates in either Talairach (Talairach and Tournoux, 1989) or Montreal Neurological Institute (MNI) standardized space were reported; (3) auditory perception, instead of reading or production tasks were utilized; (4) brain activations for attentive judgement tasks were compared with those for passive listening tasks, or attentive listening tasks of other conditions, or silent baseline. The attentive judgement tasks were chosen in order to explicitly dissociate the neural processes of different speech elements.

Since one particular study may contain experimental contrasts suited for different conditions, or may involve multiple contrasts for one condition, a secondary contrast-wise grouping process was implemented. Contrasts were retrieved and re-grouped into different conditions. Afterwards, lexical tone perception studies were divided into two conditions according to language background: lexical tone perception by native tonal language speakers (tonal tone, *n* = 12) and lexical tone perception by native non-tonal language speakers (non-tonal tone, *n* = 7). Prosody papers were separated into two conditions according to the length of elements: word-level prosody (word prosody, *n* = 15) and sentence-level prosody (sentence prosody, *n* = 25). Phoneme perception contrasts remained one condition (*n* = 14). Hence, five conditions of speech elements were identified (see Table 2 for details).

**Table 2:** Details of studies recruited in the meta-analysis.

**Study**	**Experimental task**	**Baseline task**	**No./Language of participants (sex, age)**	**Source**	**No. of Foci**
**LEXICAL TONE: TONAL**					
Gandour et al., 2000	Discrimination judgement of Thai tones	Silence	5/Thai (3F, mean 25.2 yrs)	Table 2	5
Gandour et al., 2000	Discrimination judgement of Thai tones	Silence	5/Chinese (2F, mean 25.4 yrs)	Table 2	6
Gandour et al., 2002	Discrimination judgement of Thai tones	Passive listening to hums	10/Thai (5F, mean 25.8 yrs)	Table 2	1
Gandour et al., 2003a	Discrimination judgement of Mandarin tones	Passive listening to hums	10/Mandarin (5F, mean 27.3 yrs)	Table 3	2
Hsieh et al., 2001	Discrimination judgement of Mandarin tones	Passive listening to speech contour	10/Mandarin (4F, mean 24.9 yrs)	Table 3	5
Klein et al., 2001	Discrimination judgement of Mandarin tones	Silence	12/Mandarin (6F, not provided)	Table 1	13
Li et al., 2003	Matching judgement of Mandarin tones	Syllable discrimination	12/Mandarin (6F, 23–32 yrs)	Table 2	5
Li et al., 2010	Matching judgement of Mandarin tones (random position)	Matching judgement of Mandarin tones (fixed position)	12/Mandarin (6F, 23–32 yrs)	Table 2	5
Nan and Friederici, 2013	Tone congruity judgment of Mandarin phrases	Tone congruity judgment of musical phrases	18/Mandarin (18F, 20.8 yrs)	Table 1	6
Wong et al., 2004	Discrimination judgement of Mandarin tones	Passive listening to Mandarin words	7/Mandarin (0F, 18–32 yrs)	Table 1	14
Zhang et al., 2016	Discrimination judgement of Cantonese tones (deviant tones)	Discrimination judgement of Mandarin tones (same tones)	19/Cantonese (12 F, 19.6–24.4 yrs)	Table 4	6
Zhang et al., 2017	Discrimination judgement of Cantonese tones	Discrimination judgement of musical tones	11/Cantonese (9F, 18.8–28.8 yrs)	Table 3	1
**LEXICAL TONE: NON–TONAL**					
Gandour et al., 2000	Discrimination judgement of Thai tones	Silence	5/English (2F, mean 24.6 yrs)	Table 2	6
Gandour et al., 2003a	Discrimination judgement of Mandarin tones	Passive listening to hums	10/English (5F, mean 26 yrs)	Table 3	5
Hsieh et al., 2001	Discrimination judgement of Mandarin tones	Passive listening to speech contour	10/English (5F, mean 25.6)	Table 3	3
Klein et al., 2001	Discrimination judgement of Mandarin tones	Silence	12/English (6F, not provided)	Table 1	17
Wang et al., 2003	Identification of Mandarin tones	Rest	6/English (4F, not provided)	Table 3	7
Wong et al., 2004	Discrimination judgement of Mandarin tones	Passive listening to Mandarin words	7/English (0F, 18–27 yrs)	Table 2	25
Wong et al., 2007	Discrimination judgement of Mandarin tones	Discrimination judgement of sinusoids	17/English (10F, 18–26 yrs)	Table 2	2
**PHONEME**					
Burton and Small, 2006	Discrimination judgement of English phoneme	Discrimination judgement of tone	10/not provided (8F, 20–50 yrs)	Table 3	4
Chevillet et al., 2013	Discrimination judgement of between-category phonemes	Discrimination judgement of within-category phonemes	14/English (6F, 18–32 yrs)	Table 1	16
Gandour et al., 2000	Discrimination judgement of Thai consonants	Rest	5/Thai (3F, mean 25.2 yrs)	Table 4	6
Gandour et al., 2000	Discrimination judgement of Thai consonants	Rest	5/Chinese (2F, mean 25.4 yrs)	Table 4	5
Gandour et al., 2000	Discrimination judgement of Thai consonants	Rest	5/English (2F, mean 24.6 yrs)	Table 4	6
Hsieh et al., 2001	Discrimination judgement of Chinese consonants	Passive listening to filtered speech contour	10/Chinese (4F, mean 24.9 yrs)	Table 4	5
Hsieh et al., 2001	Discrimination judgement of Chinese consonants	Passive listening to filtered speech contour	10/English (5F, mean 25.6 yrs)	Table 4	3
Obleser et al., 2006	Discrimination judgement of German vowels	Noise	13/not provided (5F, 26–36 yrs)	Table 2	5
LoCasto et al., 2004	Discrimination judgement of consonants	Discrimination judgement of tones	20/English (10F, 22–47 yrs)	Table 3	13
Rimol et al., 2005	Discrimination judgement of Norwegian consonants	Noise	17/not provided (0F, 20–28 yrs)	Table 2	1
Rogers and Davis, 2017	Discrimination judgement of consonants	Rest	24/not provided (14F, 18–45 yrs)	Table 1	6
Wolmetz et al., 2011	Discrimination judgement of between-category phonemes	Discrimination judgement of within-category phonemes	8/not provided (6F, 19–27 yrs)	Table 2	14
Zaehle et al., 2008	Discrimination judgement of consonants	Discrimination judgement of non-speech stimuli	16/Swiss-German (not provided, 22–36 yrs)	Table 1	6
Zatorre et al., 1996	Discrimination judgement of phonemes	Passive listening to noise	10/not provided (6F, not provided)	Table 6	6
**WORD PROSODY**					
Bach et al., 2008	Processing of emotional word (various tasks)	Processing of neutral word (various tasks)	16/not provided (8F, 22.1–29.9 yrs)	Table 1	9
Belyk and Brown, 2016	Emotion judgement of mono-syllables	Rest	16/not provided (10F, not provided)	Table 2	26
Brück et al., 2011	Identification of emotional words	Identification of neutral words	24/not provided (12F, 19–33 yrs)	Table 2	4
Ethofer et al., 2009	Processing of emotional words (various tasks)	Processing of neutral words (various tasks)	24/not provided (12F, mean 26.3 yrs)	Table 2	9
Frühholz et al., 2012	Processing of emotional words (various tasks)	Processing of neutral words (various tasks)	17/French (14F, 20–38 yrs)	SI Table 2	7
Gandour et al., 2004	Discrimination judgement of intonation of one syllable pair	Discrimination judgement of lexical tone of one syllable pair	10/Chinese (10F, not provided)	Table 2	3
Imaizumi et al., 1997	Discrimination judgement of emotional words	Mean reformatted MRI	6/not provided (not provided, 18–25 yrs)	Table 2	12
Kanske and Kotz, 2011	Negative words (sound location discrimination)	Neutral words (sound location discrimination)	23/German (10F, mean 25.1 yrs)	Table 2	3
Klein et al., 2011	Discrimination judgement of word prosodies	Discrimination judgement of phonemes	24/German (12F, mean 28.2 yrs)	Table 3	6
Kreitewolf et al., 2014	Discrimination judgement of word intonations	Discrimination judgement of speaker genders	17/not provided (9F, 22–34 yrs)	Table 1	15
Mothes-Lasch et al., 2012	Angry bi-syllabic nouns (unrelated task)	Neutral bi-syllabic nouns	28/not provided (21F, 18–34 yrs)	Results	1
Péron et al., 2015	Emotion judgement of emotional pseudo-words	Emotion judgement of neutral pseudo-words	15/French (12F, mean 25.12 yrs)	Table 1	14
Quadflieg et al., 2008	Processing of emotional words (various tasks)	Processing of neutral words (various tasks)	12/not provided (6F, mean 23.25 yrs)	Table 3	11
Sammler et al., 2015	Linguistic prosody judgement of words	Phoneme judgement of words	23/English (10F, 24.3–27.1 yrs)	Table 1	16
Sander et al., 2005	Angry pseudo-words (gender discrimination)	Neutral pseudo-words (gender discrimination)	15/not provided (7F, 19.8–29 yrs)	Table 1	8
**SENTENCE PROSODY**					
Alba-Ferrara et al., 2011	Classification of emotional prosodies (emotional)	Classification of emotional prosodies (neutral)	19/not provided (0F, 18–51 yrs)	Table 1	12
Beaucousin et al., 2011	Categorization of emotional sentences	Categorization of sentence gramma	23/French (12F, 20.7–26.7 yrs)	Table 2	23
Beaucousin et al., 2006	Classification of natural emotional sentences	Classification of artificial non-emotional sentences	23/French (12F, 20.3–26.3 yrs)	Table 3	20
Buchanan et al., 2000	Detection of emotional word targets	Detection of emotional phoneme targets	10/not provided (0F, 22–40 yrs)	Table 1	3
Castelluccio et al., 2016	Angry prosody sentences (unrelated judgement)	Neutral prosody sentences (unrelated judgement)	8/English (5F, 18–30 yrs)	Table 1	8
Doherty et al., 2004	Intonation judgement of sentences (question)	Intonation judgement of sentences (statement)	11/English (7F, 18–26 yrs)	Table 1	6
Escoffier et al., 2013	Judgement of emotional prosodies	Judgement of musical prosodies	16/not provided (7F, 18–26 yrs)	Table 2	5
Ethofer et al., 2006	Judgement of emotional prosodies	Judgement of emotional word contents	24/German (13F, mean 24.4 yrs)	Table 1	3
Ethofer et al., 2012	Emotional prosody (speaker gender judgement)	Neutral prosody (speaker gender judgement)	22/not provided (13F, 18.6–34 yrs)	Table 1	2
Gandour et al., 2003b	Judgement of intonations	Passive listening to speech	10/Chinese (5F, mean 26.1 yrs)	Table 2	8
Gandour et al., 2003b	Judgement of intonations	Passive listening to speech	10/English (5F, mean 28 yrs)	Table 2	15
Gandour et al., 2003b	Judgement of emotions	Passive listening to speech	10/Chinese (5F, mean 26.1 yrs)	Table 2	7
Gandour et al., 2003a	Discrimination judgement of intonations	Passive listening to speech	10/Chinese (5F, mean 27.3 yrs)	Table 3	4
Gandour et al., 2003a	Discrimination judgement of intonations	Passive listening to speech	10/English (5F, mean 26 yrs)	Table 3	5
Gandour et al., 2004	Discrimination judgement of intonations	Discrimination judgement of lexical tones	10/Chinese (10F, not provided)	Table 2	2
George et al., 1996	Emotion judgement of sentences	Active listening to sentences	13/not provided (5F, mean 28.5 yrs)	Table	2
Heisterueber et al., 2014	Discrimination judgement of suprasegmental/prosodic elements	Discrimination judgement of segmental/phonetic elements	25/German (9F, mean 28.8 yrs)	Table 3	15
Kotz et al., 2003	Emotion judgement of emotional sentences	Emotion judgement of neutral sentences	12/German (8F, 22–29 yrs)	Table 3	10
Kreitewolf et al., 2014	Discrimination judgement of sentence intonations	Discrimination judgement of verbs in sentences	17/not provided (10F, 20–29 yrs)	Table 1	22
Kristensen et al., 2013	Sentences with focused stress (semantic judgement task)	Sentences without focused stress (semantic judgement task)	24/Dutch (18F, 18–24 yrs)	Table 5	22
Leitman et al., 2010	Emotion judgement of emotional sentences	Emotion judgement of neutral sentences	19/not provided (0F, 23–33 yrs)	Table 2	14
Mitchell and Ross, 2008	Emotion judgement of emotional sentences	Rest	16/not provided (13F, 18–35 yrs)	Table 1	11
Perrone-Bertolotti et al., 2013	Sentences with focused stress (unrelated judgement task)	Sentences without focused stress (unrelated judgement)	24/French (12F, 19–34 yrs)	Table 2	10
Rota et al., 2008	Judgement of emotional prosodic sentences	Rest	10/German (0F, 24–38 yrs)	Table 1	9
Wildgruber et al., 2005	Identification of emotional sentences	Rest	10/not provided (5F, 21–33 yrs)	Table 1	17

### Activation likelihood estimation

Coordinate-based quantitative meta-analyses of neuroimaging results were performed using Ginger ALE 2.3.6 software package on the BrainMap website (www.brainmap.org/ale). The MNI coordinates were transformed into Talairach space using icbm2tal tool (Lancaster et al., 2007). ALE computes consistent activation foci by modeling probability distribution of activation at given coordinates against null distributions of group wise random spatial correlation (Eickhoff et al., 2009, 2012). In the current study, a more updated random effect Turkeltaub Non-Additive ALE method was used, which minimizes within-experiment and within-group effects by limiting probability values of neighboring foci from the same experiment (Turkeltaub et al., 2012).

Cluster-level inference was used to identify brain areas consistently recruited during perception of each condition. For protection against alterations of clusters due to small sample sizes (10–20 experiments as the current study), results were reported using an uncorrected *p* < 0.001 with cluster volume ≥540 mm^3^ as suggested (Grosbras et al., 2012). In addition, a false discovery rate (FDR, Laird et al., 2005) corrected *p* < 0.05 with an uncorrected *p* < 0.001 and minimum volume of 100 mm^3^ was used to show more stringent results as supplements.

Note that, this meta-analysis recruited studies containing different baseline conditions (silence, passive listening, and attentive listening), which may engage discrepant cognitive processes such as acoustic-phonetic analysis, lexical comprehension, attention, and manual responses. It is impossible to run ALE analyses on individual baseline conditions due to the sample size limitation. However, in order to exclude the possibility that an activation in a particular region was driven by a specific baseline contrast, foci contributions from each of the three types of baseline contrasts to each of the four groups of activation clusters were investigated. Clusters were grouped into left/right ventral (temporal lobe) and left/right dorsal (frontal and parietal lobes) streams for comparisons (see Figure S1).

Moreover, standard lateralization index (SLI) was calculated to identify the hemispheric asymmetry of activations in each condition (Dietz et al., 2016).

$$\begin{array}{l} \text{SLI}=\;\frac{Left\;Active\;Volumes\;-\;Right\;Active\;Volumes}{Left\;Active\;Volumes\;+\;Right\;Active\;Volumes} \end{array}$$

The difference between the volumes of the left and right activated clusters were divided by the sum of volumes of activated clusters in each hemisphere. The sign of SLI indicates the direction of lateralization, and it has been suggested that a SLI with an absolute value higher than 0.1 indicates asymmetry, while that between 0 and 0.1 indicates bilateral activation (Szaflarski et al., 2006).

Then, conjunction and contrast analyses were performed to determine whether various conditions yielded discrepant patterns of neural responses. Conjunction images reveal the co-activated areas between conditions, and contrast images show unique regions recruited for perception of particular condition. Pairwise conjunction and contrast analyses were implemented between tonal tone and each of the other conditions (i.e., non-tonal tone, phoneme, word prosody, and sentence prosody). Here, contrasts were calculated using a voxel-wise minimum statistic (Nichols et al., 2005; Eickhoff et al., 2011), which ascertained the intersection between the individually thresholded meta-analysis results and produced a new thresholded ALE image (uncorrected *p* < 0.05, with 10,000 permutations and minimum volume of 100 mm^3^). This procedure was conducted on both uncorrected (uncorrected *p* < 0.001, minimum volume = 540 mm^3^, see **Figure 5**, Figure S4 and, **Tables 4**,**5**) and corrected (FDR-corrected *p* < 0.05, minimum volume = 100 mm^3^, see Figure S4 and Tables S2, S3) ALE results, respectively.

To visualize the results, multiple software packages were utilized. BrainNet software was used to demonstrate foci (Xia et al., 2013). Volume images as well as 3D displays were generated by Mango software (http://ric.uthscsa.edu/mango/download.html), utilizing ch2better template from Mricron package (https://www.nitrc.org/projects/mricron). The ALE maps were also projected onto a cortical inflated surface template using FreeSurfer, and visualized by FreeView (http://www.freesurfer.net/).

## Results

### Neural substrates of each condition

**Figure 2:**
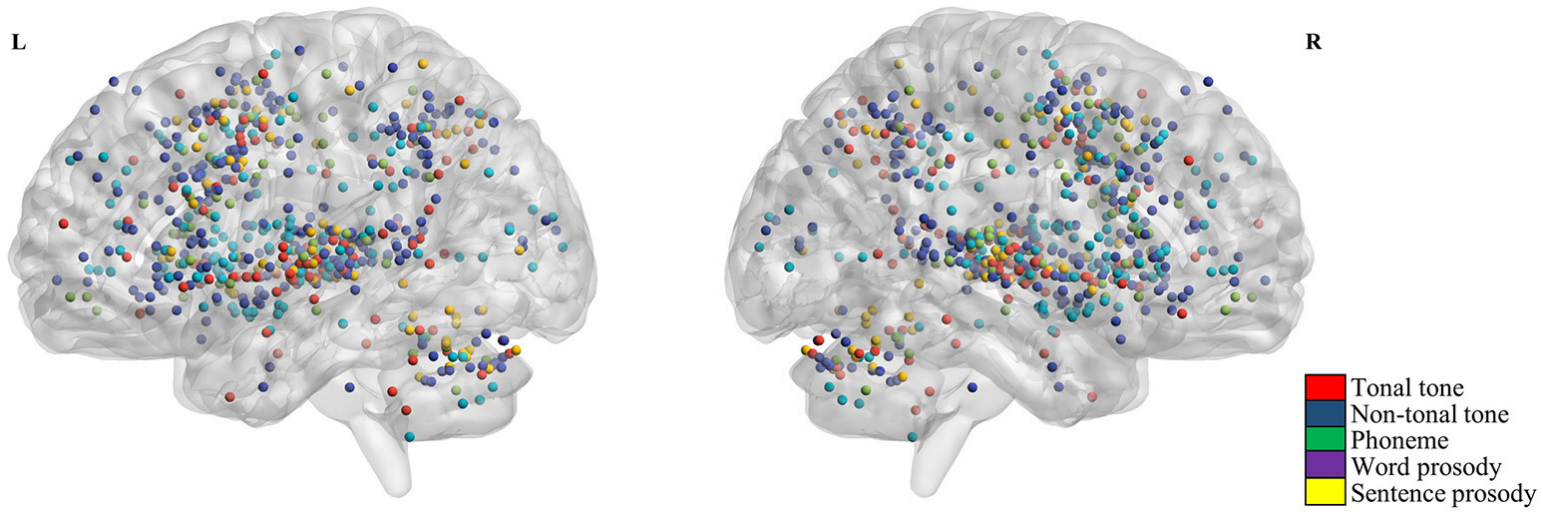
Activation foci from selected contrasts. Red, blue, green, violet, and yellow dots represent foci from tonal tone, non-tonal tone, phoneme, word prosody and sentence prosody, respectively. Across conditions, foci were widely distributed in bilateral temporal, frontal, parietal regions, and the cerebellum.

**Figure 3:**
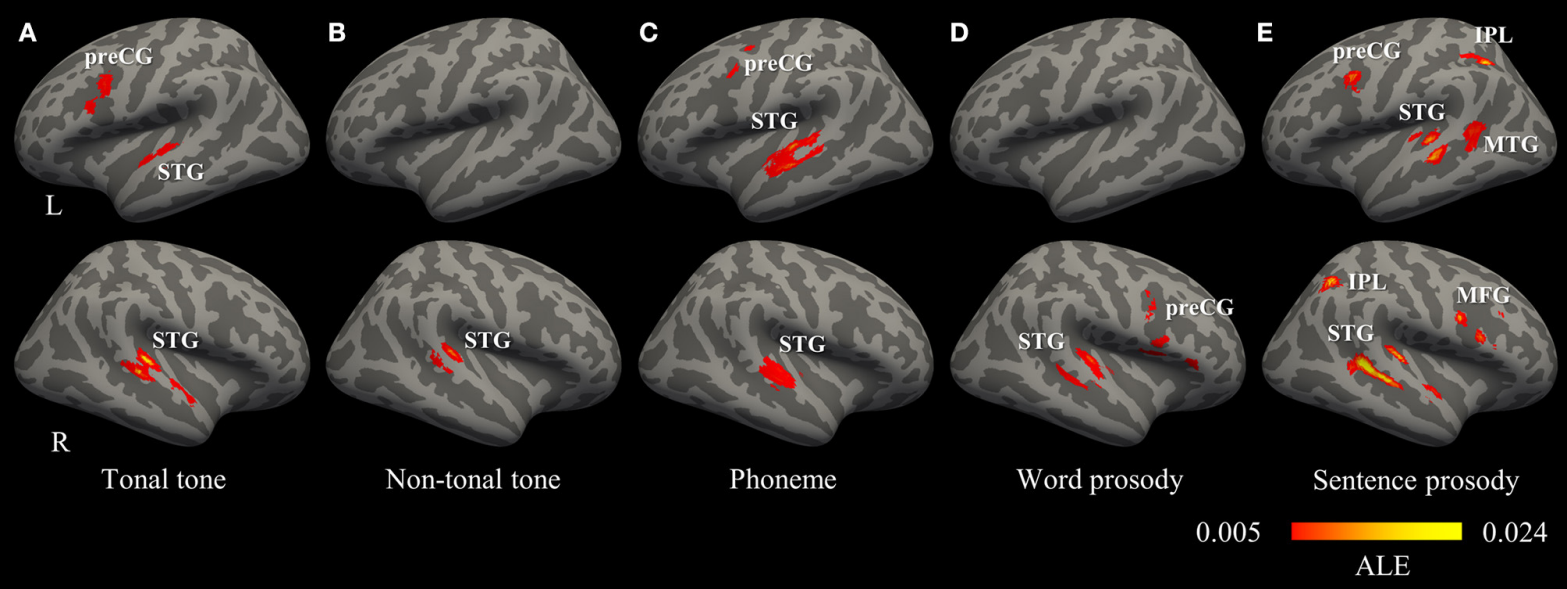
Convergence of activations in each condition (uncorrected *p* < 0.001, minimum cluster = 540 mm^3^). **(A–E)** Regions consistently activated by the perception of tonal tone, non-tonal tone, phoneme, word prosody, and sentence prosody, respectively. IPL, inferior parietal lobule; MFG, middle frontal gyrus; MTG, middle temporal gyrus; preCG, precentral gyrus; STG, superior temporal gyrus.

Figure 2 shows the individual foci used in the meta-analyses for each condition. Regardless of conditions, foci were widely distributed in bilateral temporal, frontal, parietal lobes and the cerebellum. Brain regions consistently activated by each condition were shown in Figure 3 and Table 3 (uncorrected, see Figure S2 for volumetric sections of activations in each condition).

**Table 3:** Brain regions consistently activated in each condition (uncorrected *p* < 0.001, minimum cluster = 540 mm^3^).

**Brain region**	**BA**	**Peak talairach coordinates**			**Max. ALE (× 10^−2^)**	**Volume (mm^3^)**	**Ratio of studies**
		**x**	**y**	**z**			
**TONAL TONE**							
R Superior Temporal Gyrus	22	58	−24	4	2.63	2,440	0.50
L Superior Temporal Gyrus	41	−58	−18	8	1.54	2,104	0.42
R Cerebellum	NA	2	−64	−26	1.49	1,960	0.50
L Medial Frontal Gyrus	6	0	18	44	1.31	1,936	0.50
L Precentral Gyrus	9	−40	4	32	1.86	1,832	0.42
R Superior Temporal Gyrus	22	56	−4	0	1.30	1,064	0.33
**NON-TONAL TONE**							
R Superior Temporal Gyrus	41	56	−26	10	2.07	1,808	0.57
**PHONEME**							
L Superior Temporal Gyrus	22	−56	−16	0	2.17	4,376	0.79
R Superior Temporal Gyrus	22	60	−18	2	1.69	2,256	0.57
L Precentral Gyrus	6	−38	0	42	1.21	576	0.21
**WORD PROSODY**							
R Precentral Gyrus	44	46	10	10	1.58	3,648	0.60
R Superior Temporal Gyrus	22	48	−22	4	1.92	2,512	0.53
L Putamen	NA	−24	10	6	1.49	1,080	0.27
L Amygdala	NA	−20	−10	−12	1.63	752	0.27
**SENTENCE PROSODY**							
R Superior Temporal Gyrus	22	46	−36	4	2.92	4,208	0.56
R Middle Frontal Gyrus	9	48	14	30	3.06	2,976	0.44
L Medial Frontral Gyrus	6	0	14	48	2.43	2,840	0.40
L Middle Temporal Gyrus	21	−56	−28	2	2.25	2,744	0.36
L Precentral Gyrus	6	−42	4	34	2.38	2,048	0.32
R Inferior Parietal Lobule	40	34	−54	44	3.11	1,744	0.32
L Inferior Parietal Lobule	40	−34	−52	34	2.34	1,224	0.28
L Middle Temporal Gyrus	21	−54	−46	6	1.75	1,152	0.24
R Superior Temporal Gyrus	38	52	−2	−6	1.63	808	0.16

The total number of foci for tonal tone was 69, with 40 of them located in the left hemisphere. Perception of tonal tone was associated with peak activations in bilateral STG, the left preCG, the left medial frontal gyrus (MeFG) and the right cerebellum (Figure 3A and Figure S2). In contrast, non-tonal tone revealed 65 foci, 36 of which were located in the left hemisphere, yielding peak activity only in the right STG (Figure 3B and Figure S2).

For phoneme perception, 96 foci were included with 58 of them located in the left hemisphere, and consistent peak activations were observed in bilateral STG and the left preCG (Figure 3C and Figure S2).

Foci for word prosody were 144, 66 of which resided in the left hemisphere. Perception of word prosody showed consistent peak activities in the right STG, the right preCG, the left putamen and the left amygdala (Figure 3D and Figure S2). Sentence prosody included 255 foci, with 127 of them spread in the left hemisphere. Prosody perception at the sentence level yielded peak activations in bilateral STG, the right temporal pole, the left middle temporal gyrus (MTG), the left preCG, the left MeFG, the right middle frontal gyrus (MFG), and bilateral IPL (Figure 3E and Figure S2).

Thus, convergent activations in bilateral auditory cortices were found in tonal tone, phoneme and sentence prosody, whereas non-tonal tone and word prosody consistently recruited the right auditory cortex only. Moreover, a left asymmetric activation in superior and middle temporal lobes was revealed for phoneme (left volume 4,376 mm^3^ > right volume 2,256 mm^3^, SLI = 0.32), while the opposite pattern was shown for tonal tone (right volume 3,504 mm^3^ > left volume 2,104 mm^3^, SLI = −0.25) and sentence prosody (right volume 5,016 mm^3^ > left volume 3,896 mm^3^, SLI = −0.13). Additionally, consistent activations of the preCG were found in the left hemisphere for tonal tone and phoneme, in the right hemisphere for word prosody, and bilaterally for sentence prosody. Note that FDR correction did not substantially change the results, except that the left preCG was not activated for phoneme perception and no activation was found for word prosody (Table S1).

Moreover, although the baseline condition varied in different studies, most of the cluster groups in bilateral ventral and dorsal streams were contributed by foci from each type of baseline contrasts (rest, passive listening, and active listening, Figure S1). Although *post-hoc* statistical tests on foci contributions were not conducted due to the sample size limitation, it is clear that the activation patterns were not driven by specific baseline conditions.

### Overlap between patterns of activations

**Figure 4:**
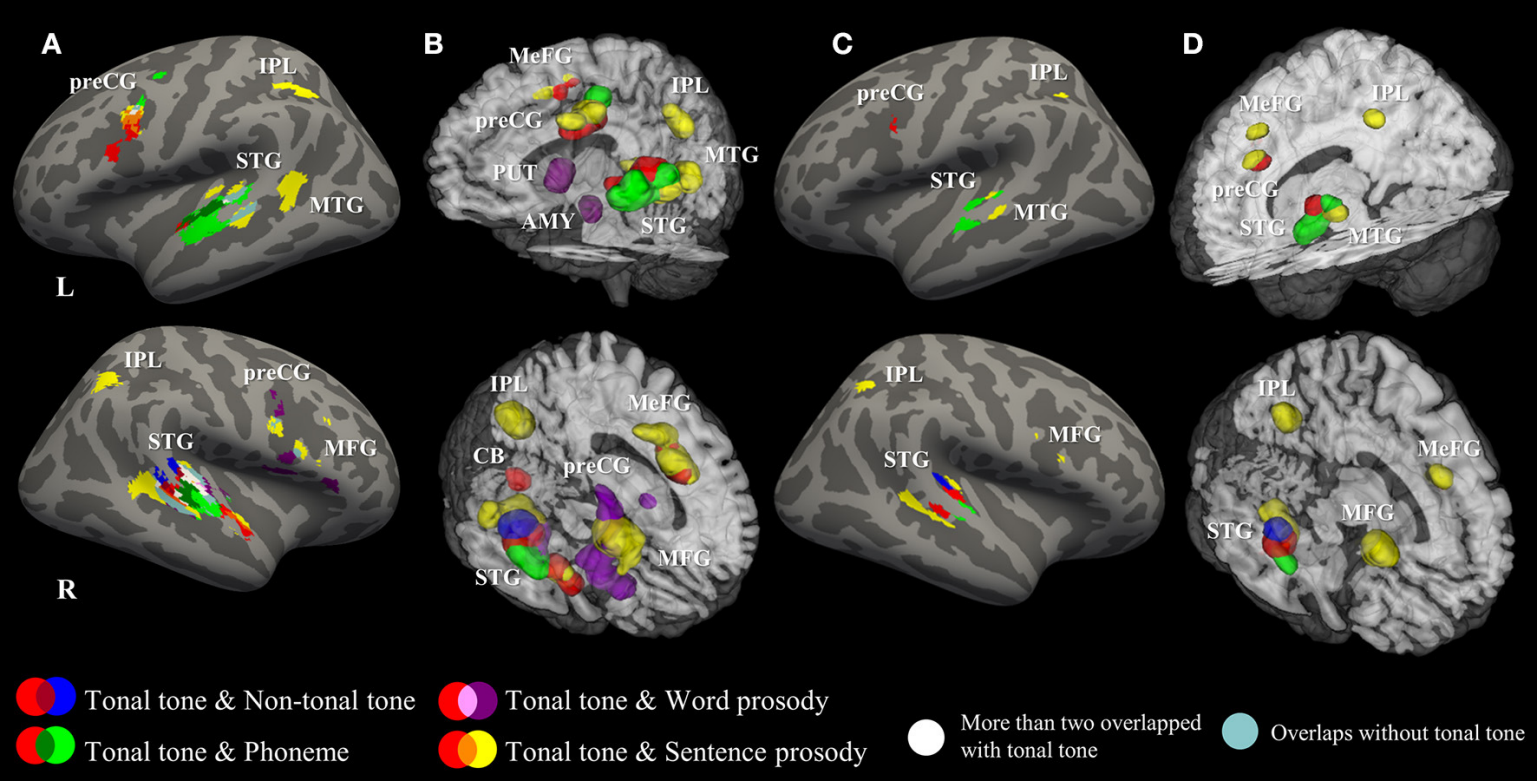
Surface and 3D Rendering maps showing overlaid ALE statistics for all conditions. **(A,B)** uncorrected *p* < 0.001, minimum cluster = 540 mm^3^. **(C,D)** FDR-corrected *p* < 0.05, minimum cluster = 100 mm^3^. AMY, amygdala; CB, cerebellum; IPL, inferior parietal lobule; MFG, middle frontal gyrus; MeFG, medial frontal gyrus; MTG, middle temporal gyrus; preCG, precentral gyrus; PUT, putamen; STG, superior temporal gyrus.

Figure 4 illustrates the spatial relationship of activations associated with different conditions. Hierarchical organizations of representations were shown in bilateral STG and in the left preCG. In the left STG, activations for tonal tone and phoneme were located anterior to that for sentence prosody. In the right STG, an anterior-lateral to posterior-medial oblique axis of successive activations for segmental elements (phoneme), syllabic elements (tonal tone, non-tonal tone, and word prosody resided in more anterior, superior, and inferior-medial portions, respectively) and sentence prosody (surrounded from medial to posterior then to lateral-inferior portions) was revealed (Figures 4A,B). Such an anterior-posterior (left STG) or anterior-lateral to posterior-medial (right STG) gradient of representations in bilateral STG with increasing element timescale became more obvious after FDR correction (Figures 4C,D). Before FDR-correction, an additional activation shared by lexical tone and sentence prosody was located in the right anterior STG (aSTG). In addition, area consistently activated for tonal tone in the left preCG largely overlapped with that of prosody and was ventral to that of phoneme (Figures 4A,B).

**Figure 5:**
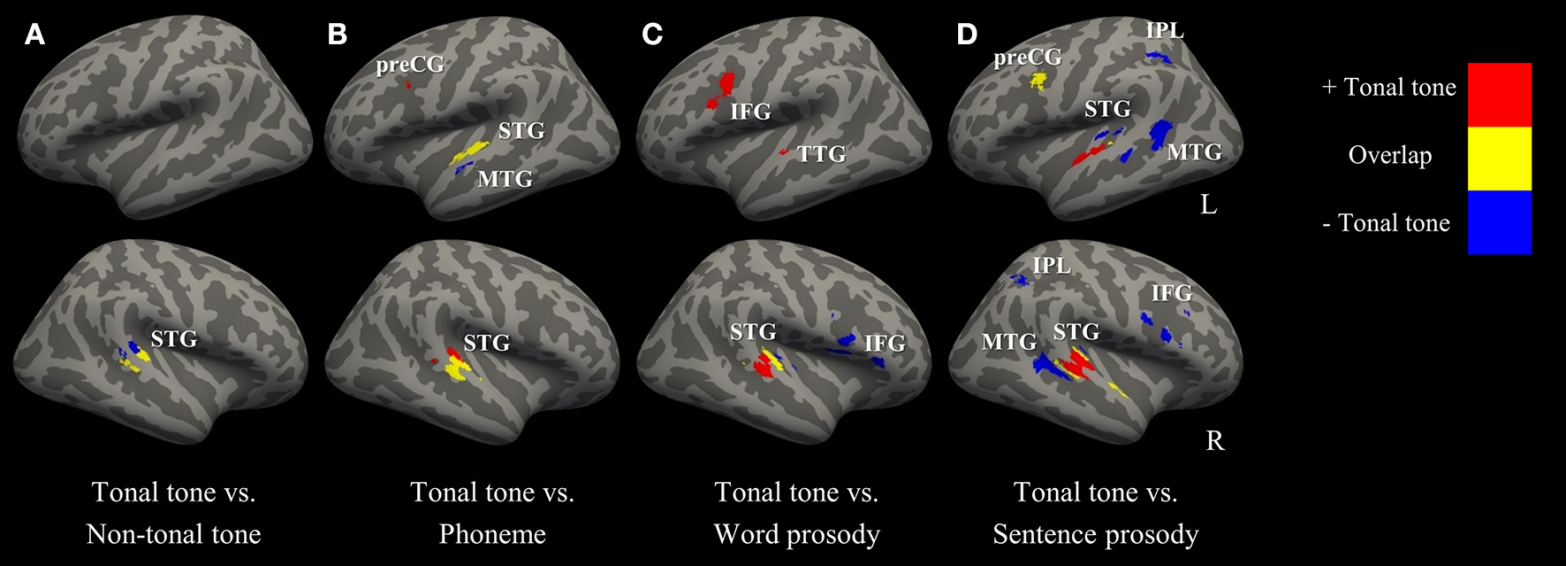
Conjunction and contrast maps between tonal tone and other conditions (uncorrected *p* < 0.001, minimum cluster = 100 mm^3^). **(A–D)** comparisons of tonal tone with non-tonal tone, phoneme, word prosody, and sentence prosody, respectively. Red: regions uniquely recruited in tonal tone compared with one of the other conditions; yellow: regions coactivated in tonal tone and one of the other conditions; blue: regions specifically engaged in one of the other conditions compared with tonal tone. IFG, inferior frontal gyrus; IPL, inferior parietal lobule; MTG, middle temporal gyrus; preCG, precentral gyrus; STG, superior temporal gyrus; TTG, transverse temporal gyrus.

Figure 5 (surface maps), Figure S3 (3D maps), and Table 4 show uncorrected regions that were co-activated by tonal tone and other conditions. The conjunction analyses between tonal tone and non-tonal tone (Figure 5A) or word prosody (Figure 5C) revealed overlap in the right STG. The conjunction analysis between tonal tone and phoneme yielded bilateral overlaps in the STG (Figure 5B). The conjunction analysis between tonal tone and sentence prosody showed co-activation in bilateral STG (the right STG overlap extended into the anterior temporal pole) and in the left preCG (Figure 5D). After FDR correction, tonal tone only shared activations with non-tonal tone in the right STG and with sentence prosody in the left preCG (Figure S4 and Table S2).

**Table 4:** Co-activated regions for tonal tone and other conditions based on uncorrected ALE results (uncorrected *p* < 0.001, minimum cluster = 100 mm^3^).

**Brain region**	**BA**	**Peak talairach coordinates**			**Max. ALE (× 10^−2^)**	**Volume (mm^3^)**
		**x**	**y**	**z**		
**TONAL TONE ∩ NON-TONAL TONE**						
R Superior Temporal Gyrus	41	56	−24	8	1.64	992
**TONAL TONE ∩ PHONEME**						
L Superior Temporal Gyrus	41	−56	−18	4	1.39	1,112
R Superior Temporal Gyrus	41	60	−20	2	1.64	944
**TONAL TONE ∩ WORD PROSODY**						
R Superior Temporal Gyrus	41	52	−22	6	1.31	736
**TONAL TONE ∩ SENTENCE PROSODY**						
L Medial Frontal Gyrus	6	0	18	44	1.31	1,360
L Precentral Gyrus	9	−40	4	32	1.86	872
R Superior Temporal Gyrus	22	54	−28	6	1.30	504
R Superior Temporal Gyrus	22	56	−4	0	1.30	400
L Superior Temporal Gyrus	41	−58	−22	8	1.28	264

### Contrast between patterns of activations

Figure 5 (surface maps), Figure S3 (3D maps), and Table 5 display results from the contrast analyses on uncorrected ALE maps. Tonal tone yielded more consistent patterns of activations in the left preCG, the right pSTG and the right cerebellum than phoneme (Figure 5B); in the left transverse temporal gyrus, the right STG, the left inferior frontal gyrus (IFG), the left MeFG and the left cerebellum than word prosody (Figure 5C); and in bilateral aSTG and the right cerebellum than sentence prosody (Figure 5D). In contrast, tonal tone revealed less consistent patterns of activations in the right pSTG than non-tonal tone (Figure 5A); in the left anterior MTG than phoneme (Figure 5B); in the right STG and the right IFG than word prosody (Figure 5C); and in bilateral posterior STG and MTG, bilateral IPL, and the right IFG than sentence prosody (Figure 5D).

**Table 5:** Brain regions revealed by contrasting tonal tone with other conditions based on uncorrected ALE results (uncorrected *p* < 0.001, minimum cluster = 100 mm^3^).

**Brain Region**	**BA**	**Peak Talairach Coordinates**			**Z Score**	**Volume (mm^3^)**
		**x**	**y**	**z**		
**TONAL TONE > NON-TONAL TONE**	None
**NON-TONAL TONE > TONAL TONE**						
R Superior Temporal Gyrus	22	62	−32	10	2.54	728
**TONAL TONE > PHONEME**						
R Cerebellum	NA	2	−66	−20	2.40	1,256
R Superior Temporal Gyrus	41	56	−26	12	2.41	856
L Middle Frontal Gyrus	9	−42	12	32	2.04	184
**PHONEME > TONAL TONE**						
L Middle Temporal Gyrus	21	−62	−4	−4	1.98	296
**TONAL TONE > WORD PROSODY**						
L Cerebellum	NA	2	−64	−21	2.63	1,904
L Inferior Frontal Gyrus	9	−40	6	26	2.57	1,176
L Medial Frontal Gyrus	8	−4	18	46	2.52	1,112
R Superior Temporal Gyrus	22	62	−26	2	2.48	784
L Transverse Temporal Gyrus	42	−59	−16	12	2.38	712
**WORD PROSODY > TONAL TONE**						
R Inferior Frontal Gyrus	44	45	13	16	3.29	2,704
L Parahippocampal Gyrus	NA	−28	−7	−16	2.48	688
R Superior Temporal Gyrus	22	44	−22	4	1.82	240
**TONAL TONE > SENTENCE PROSODY**						
R Cerebellum	NA	2	−67	−21	3.54	1,704
R Superior Temporal Gyrus	22	60	−21	8	3.72	1,632
L Superior Temporal Gyrus	22	−54	−12	3	2.62	1,192
**SENTENCE PROSODY > TONAL TONE**						
R Inferior Frontal Gyrus	45	48	23	23	3.89	2,760
R Middle Temporal Gyrus	22	48	−44	5	2.77	1,920
L Middle Temporal Gyrus	22	−53	−42	6	2.99	1,152
R Inferior Parietal Lobule	40	34	−48	42	2.48	1,104
L Superior Temporal Gyrus	41	−44	−32	8	3.01	856
L Inferior Parietal Lobule	40	−36	−44	44	2.05	568

After FDR correction, tonal tone showed stronger convergent activation in the right STG than phoneme and sentence prosody (Figures S4B,D and Table S3). Meanwhile, non-tonal tone elicited stronger activation than tonal tone in a right STG subregion posterior to their co-activated site (Figure S4A and Table S3). Compared with tonal tone, sentence prosody showed consistently stronger activations in the STG (posterior to the region where tonal tone had stronger activation), IFG and IPL in the right hemisphere (Figure S4D and Table S3).

## Discussion

The current meta-analysis aimed at identifying discrepant as well as shared neural systems underlying perception of lexical tone, phoneme, and prosody. Results are discussed based on the dual-stream model of speech processing (Hickok and Poeppel, 2007), focusing on the hemispherical asymmetry and the gradient of representations in each stream.

### Ventral stream of lexical tone perception

#### Hemispherical asymmetry

Auditory regions consistently recruited for phoneme perception asymmetrically resided in the left hemisphere, whereas the opposite pattern was found for other linguistic elements. This is consistent with the model of spectrotemporal resolution (Zatorre et al., 2002) and the AST model (Poeppel, 2003) that speech information in short and long temporal windows are predominantly processed in the left and right auditory cortex, respectively. Importantly, only native tonal language speakers consistently recruited the left STG in lexical tone perception, an area also involved in phoneme perception, supporting the notion that language experience shapes lexical tone as a phonetic feature in defining lexical meaning (Gandour et al., 2003a; Gu et al., 2013). Moreover, regardless of language background, right asymmetrical activations in the auditory ventral stream were found during lexical tone perception, which is in line with the findings from a recent meta-analysis (Kwok et al., 2017) and the fact that the right hemisphere is advantaged at processing spectrally variant sounds (Zatorre and Belin, 2001; Zatorre et al., 2002; Luo et al., 2006).

#### Gradient of representations

Representational topographies were shown in bilateral STG as a function of element timescale. That is, segmental and syllabic elements were anterior to sentence prosody in the left STG; while segmental element, syllabic elements and sentence prosody were aligned along the anterior-lateral to posterior-medial oblique axis. Differences in acoustic-phonetic features between selected speech elements (see Table 1) may account for the observed gradients of representations in auditory cortices. Specifically, phoneme is determined by the rapid transitions of the first and second formants (~200–2,500 Hz) in short time windows (~40–150 ms, 6–25 Hz). In contrast, lexical tone and prosody are defined by variations of the fundamental frequency (~80–250 Hz) that develops in longer time windows (from syllabic length to sentence-wise length, >200 ms, < 5 Hz). This corresponds to differences in neural encoding demands for rates of spectral and temporal modulation. The gradient of representations in bilateral STG (especially in the right hemisphere) is consistent with previous findings showing that the anterior and posterior STG were tuned for higher spectral and lower temporal modulation, respectively (Santoro et al., 2014; Hullett et al., 2016). The anterior-posterior hierarchy of representations in bilateral STG was in line with increasing element timescale, which resembled the findings from Lerner et al. (2011).

Moreover, the linguistic functions of speech elements may interact with their acoustic features to build the hierarchical organization of representations, which may explain the differences between gradients in bilateral STG. The left anterior STG, co-activated by tonal tone and phoneme in the current study, has been implicated in auditory word-form recognition (DeWitt and Rauschecker, 2012). Whereas, in the right STG, a clear gradient as a function of element timescale was revealed, indicating that the pattern was mainly driven by the spectrotemporal resolution of auditory cortex but less modulated by higher-level linguistic cognitions. One of the questions that need to be addressed in the field of speech perception is to what extent does perception rely on fine temporal and/or spectral structures, and how these weights are altered by the type of linguistic cues. Future studies are expected to investigate how spectrotemporal analysis of speech signals interacts with phonological and semantic representations to form the hierarchical organizations in auditory cortices.

In addition, co-activated areas between tonal tone and sentence prosody extended toward aSTG (temporal pole, BA 38) in the right hemisphere. The right aSTG has been suggested to evaluate emotions of prosody (Kotz and Paulmann, 2011; Belyk and Brown, 2013). Indeed, the current study grouped together prosody studies that required judgements of emotions and evaluations of linguistic features, which resulted in consistent activations in the emotional system (e.g., amygdala activation in word prosody). This also coincides with previous findings that the right aSTG was crucial for lexical tone processing in tonal language speakers (Ge et al., 2015). Moreover, in one latest study comparing musicians and non-musicians in a syllable-in-noise identification task, the right aSTG showed stronger functional connectivity with right auditory cortex in musicians, and this connectivity positively correlated with judgement accuracy (Du and Zatorre, 2017). This indicates the role of the right aSTG in abstract representations of suprasegmental linguistic objects, which is likely involved in the perception of lexical tone and prosody. Because the right aSTG was not activated for tonal tone and sentence prosody after FDR correction (Figures 4C,D), and has a different functional role from the posterior portion of STG which is involved in spectrotemporal analysis of speech signals (Hickok and Poeppel, 2007), it was not considered in the gradient of representations in the right STG.

### Dorsal stream of lexical tone perception

#### Hemispherical asymmetry

In the current study, tonal tone and phoneme evoked convergent activations in the left preCG, word prosody elicited consistent activations in the right preCG, whereas sentence prosody engaged consistent bilateral preCG activations. Patterns of asymmetry for the motor/premotor regions in speech perception are consistent with previous investigations using phoneme (Du et al., 2014), prosody at syllabic length (Sammler et al., 2015) and prosody at sentence level (Witteman et al., 2012; Belyk and Brown, 2013). However, different from a recent ECoG study that showed bilateral recruitment of speech motor regions during tone perception (Si et al., 2017) and a recent meta-analysis on lexical tone processing (including both perception and production tasks) showing activations in bilateral inferior frontal cortices (Kwok et al., 2017), this study only revealed consistent activation in the left premotor regions. This discrepancy might result from the small number of contrasts recruited that weakened the statistical power, or which is more likely, the less robust involvement of the right speech motor areas compared with the left ones during lexical tone perception compared with lexical tone production. Note that, this meta-analysis only recruited studies using attentive judgement tasks, which may strengthen the dorsal stream engagement and corresponding sensorimotor integration in speech perception.

One area related to the dorsal stream engagement is the cerebellum, which is implicated in the planning and execution of motor responses and internal motoric representation of speech (Hsieh et al., 2001). Here, tonal tone revealed an activation in the right cerebellum before the FDR correction. The perception of lexical tone in tonal language speakers may involve stronger articulatory rehearsal than non-tonal language speakers, which would activate the cerebellum to some extent. In addition, such an activation was contributed by six studies with five of them using passive listening or silence as the baseline. This suggests that the cerebellum activation in tonal tone was possibly driven by the execution of manual responses during judgement. However, those could not fully explain the failure to find cerebellum activation in other conditions, as all other conditions recruited a large amount of studies without judgement in baseline conditions and internal articulatory representations were revealed in the processing of phoneme and prosody as well.

Overall, our results suggest that perception of lexical tone in tonal language speakers not only recruited a bilateral temporal hierarchy but also involved a left lateralized speech motor network in the dorsal stream, a pattern that resembles phoneme perception.

#### Gradient of representations

As predicted, the activation for tonal tone largely overlapped with that for sentence prosody, but was ventral to the activation for phoneme in the left preCG. Since it is evident that phoneme perception engaged speech motor areas controlling lips and tongue in a feature-specific manner (Schomers and Pulvermüller, 2016) and different articulation organs are topographically represented in the so-called “motor strip” (Penfield and Boldrey, 1937), such a dorsal-ventral spatial distribution in the left preCG may correspond to the variant places of articulation for phoneme (lips and tongue) and prosody/lexical tone (larynx). Notably, it is unlikely that manual responses substantially contributed to the left preCG activation. Firstly, the observed preCG activation resided in the ventral portion of premotor cortex, while motor areas controlling for fingers locate in the dorsal portion of the motor/premotor strip. Secondly but more importantly, as shown in Figure S1, almost half of the foci in the left dorsal stream during speech perception were contributed by contrasts that controlled the manual response artifacts (i.e., task-related attentive listening—task-unrelated attentive listening). Thus, resembling prosody, lexical tone perception in tonal language speakers possibly recruited the laryngeal sensorimotor network, which, however, need to be confirmed by direct localization tasks in future studies.

As for the functional role, consistent activation of the left ventral premotor cortex during lexical tone perception indicates an internal model of laryngeal movements that might anticipate the pitch pattern of the speaker embedded in speech signals (Hickok and Poeppel, 2007; Rauschecker and Scott, 2009). Such predictions are suggested to be matched with auditory representations in the sensorimotor interfaces (e.g., left pSTG and IPL) to aid speech perception, particularly in challenging listening environments. Consistent activations in the pSTG and IPL were indeed observed bilaterally in sentence prosody, but these regions were not consistently activated in other tasks. This slightly blurs the picture of sensorimotor integration in lexical tone perception, presumably due to the small number of studies recruited and the ideal testing conditions for lexical tone in almost all the studies.

### Dynamic dual-stream model for lexical tone perception

**Figure 6:**
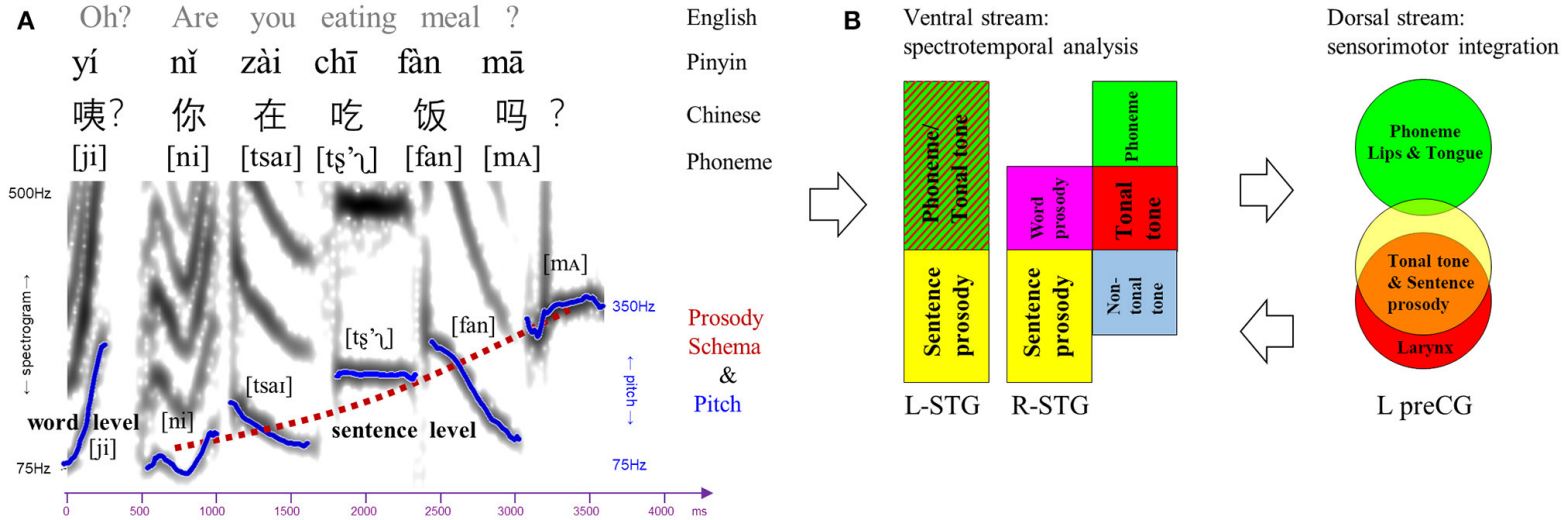
Dual-stream model for speech perception. **(A)** An example of Chinese sentence comprised of various linguistic elements (phoneme, lexical tone, word-level, and sentence-level prosody). The spectrogram and pitch contours of each lexical tone and prosody were extracted from the sentence spoken by a male Mandarin native speaker. Notably, lexical tone can bridge single vowel, double vowels (e.g., /ai/), triple vowels, or vowel and nasal consonant (e.g., /an/), although it is labeled upon single vowel in Pinyin. **(B)** Dual-stream model for speech perception in which the ventral stream is involved in spectrotemporal analysis and the dorsal stream is responsible for sensorimotor integration. Ventral stream: gradient representations of different linguistic elements in bilateral superior temporal gyrus (STG). Dorsal stream: topological representations of phoneme, tonal tone and sentence prosody in the left precentral gyrus (preCG), corresponding to different places of articulation.

In the exemplar Chinese sentence (Figure 6A), different speech elements at various timescales coincide to convey linguistic and paralinguistic information. Note that, lexical tone can bridge single vowel, double vowels (e.g., diphthong /ai/ in this example), triple vowels (e.g., triphthong /iao/), or vowel and nasal consonant (e.g., /an/ in this example), confirming its suprasegmental nature and substantive identity compared with segmental phoneme. Moreover, despite its phonemic nature by definition and suprasegmental timescale, lexical tone is distinct from segmental phoneme in terms of the place of articulation and neural network. Meanwhile, in a metaphorical description (Chao, 1968), semantic-related lexical tones fluctuate as “small waves” upon the “big wave” of pragmatic-related prosody. In spite of acoustic similarities in pitch variations, lexical tone and prosody have discrepant linguistic functions and underlying neural processes.

A dynamic dual-stream model is thus proposed based on our findings to delineate the neurocognitive processes of lexical tone perception (Figure 6B). In such a model, bilateral STG in the ventral stream are recruited to decipher the spectrotemporal information of the syllabic pitch contours embedded in incoming speech signals. Bilateral STG also demonstrate gradients of representations as a function of element timescale. In the left STG, lexical tone in tonal language speakers is processed in the anterior portion, a site involved in phonemic processing and word-form recognition (DeWitt and Rauschecker, 2012), while sentence prosody which is longer in duration than lexical tone and phoneme is analyzed in the posterior portion. In the right STG, along an anterior-lateral to posterior-medial oblique axis, the subregion that decodes lexical tone in tonal language speakers lies posterior to that for phoneme, anterior to that for lexical tone by non-tonal language speakers, and anterior as well as lateral to that for prosody. In the dorsal stream, processing of lexical tone only in tonal language speakers engages the left lateralized articulatory network. Specifically, the left preCG shows a dorsal-ventral distribution of representations for phoneme and lexical tone/prosody, likely corresponding to the differentiated places of articulation (i.e., lips/tongue vs. larynx) and associated sensorimotor mapping. Presumably, an internal model of speech motor gestures by larynx would be generated in the left ventral premotor cortex to predict and constrain the auditory representations of lexical tone in bilateral auditory cortices via feedback and feedforward projections. Such a dynamic dual-stream model coordinates the spectrotemporal analysis and sensorimotor integration in lexical tone perception.

### Limitations and expectations

Meta-analysis recruits a large amount of previous studies sharing similar topics to reduce bias from a single study. It also facilitates the comparison of neural networks yielded by different tasks and stimuli from different groups of people. However, this meta-analysis is limited for the comparatively small sample size. Hence, interpretations on hemispherical asymmetry and topological representations should be taken with caution, as clusters with relatively low ALE scores may be rejected. Moreover, this meta-analysis only recruited fMRI and PET studies, which have poor temporal resolution, therefore falls short of revealing the dynamic shift of hemispherical asymmetry of lexical tone perception from low to high levels across time. Research approaches with high spatial-temporal resolution, such as magnetoencephalography (MEG) and ECoG, are encouraged to depict the neural dynamics of lexical tone perception in the future.

## Conclusion

This meta-analysis elaborated the functional neuroanatomy of lexical tone perception, which was intermixed with that of phoneme and that of prosody in terms of hemispherical asymmetry and regional hierarchical organizations. Resembling prosody, right asymmetric activations of auditory cortices in the ventral stream were found for lexical tone regardless of language background, whereas tonal language speakers additionally recruited the left STG for parsing tone as a phonemic feature in lexical mapping. Bilateral STG also showed hierarchical organizations of representations as a function of element timescale, in which the activation for lexical tone lied between that for phoneme and that for prosody particularly in the right hemisphere. Moreover, different from a bilateral recruitment of speech motor regions in the dorsal stream for sentence prosody, a left lateralized speech motor activation was revealed for processing phoneme and lexical tone in tonal language speakers. Finally, activations in the left preCG for various speech elements corresponded to their articulatory patterns. During tone perception, tonal language speakers engaged the left preCG subregion implicated in prosody perception, consistent with the idea that stronger articulatory representations in the laryngeal sensorimotor network were achieved by tonal language speakers for parsing lexical tone. Hence, perception of lexical tone is shaped by language experience and involves a dynamic dual-stream processing. Future research with more sophisticated methods are called for delineating the dynamic and cooperative cortical organizations of speech perception in integration of different linguistic elements and for various languages, respectively.

## Author contributions

BL acquired the data, conducted the meta-analysis, contributed to the interpretation of the results and wrote the manuscript. YD designed the study, contributed to the interpretation of the results and wrote the manuscript.

### Conflict of interest statement

The authors declare that the research was conducted in the absence of any commercial or financial relationships that could be construed as a potential conflict of interest.
