# Supplementary material for: The Functional Neuroanatomy of Lexical Tone Perception: An Activation Likelihood Estimation Meta-Analysis
Source: Front Neurosci. 2018 Jul 24;12:495. doi: 10.3389/fnins.2018.00495 (PMC6066585; doi:10.3389/fnins.2018.00495)
Supplement: Supplementary file 1 [file Table_1.DOC]

**TABLE S1 | Brain regions consistently activated in each condition (FDR-corrected *p* < 0.05,** **minimum cluster = 100 mm^3^).**

| **Brain Region** | **BA** | **Peak Talairach Coordinates** | | | | | | **Max. ALE**  **(×10^-2^)** | **Volume (mm^3^)** | **Ratio of Studies** |
| --- | --- | --- | --- | --- | --- | --- | --- | --- | --- | --- |
|  |  | **x** | | | **y** | | **z** |  |  |  |
| **Tonal Tone** |  | | | | | | | | | |
| R Superior Temporal Gyrus | 22 | 58 | | | -24 | | 4 | 2.63 | 872 | 0.50 |
| L Precentral Gyrus | 9 | -40 | | | 4 | | 32 | 1.86 | 240 | 0.25 |
| L Superior Temporal Gyrus | 41 | -58 | | | -18 | | 8 | 1.54 | 168 | 0.08 |
| **Non-tonal Tone** |  | | | | | | | | | |
| R Superior Temporal Gyrus | 41 | 56 | | -26 | | 10 | | 2.07 | 488 | 0.57 |
| **Phoneme** |  | | | | | | | | | |
| L Superior Temporal Gyrus | 22 | -56 | | -16 | | 0 | | 2.17 | 800 | 0.36 |
| R Superior Temporal Gyrus | 22 | 60 | | -18 | | 2 | | 1.69 | 280 | 0.14 |
| L Superior Temporal Gyrus | 22 | -60 | | -28 | | 6 | | 1.64 | 112 | 0.07 |
| **Word Prosody** | None | | | | | | | | | |
| **Sentence Prosody** |  | | | | | | | | | |
| R Superior Temporal Gyrus | 22 | 46 | -36 | | | 4 | | 2.92 | 1328 | 0.36 |
| R Middle Frontal Gyrus | 9 | 48 | 14 | | | 30 | | 3.06 | 1136 | 0.36 |
| R Inferior Parietal Lobule | 40 | 34 | -54 | | | 44 | | 3.11 | 664 | 0.20 |
| L Medial Frontal Gyrus | 6 | 0 | 14 | | | 48 | | 2.43 | 392 | 0.16 |
| L Middle Temporal Gyrus | 21 | -56 | -28 | | | 2 | | 2.25 | 344 | 0.08 |
| L Precentral Gyrus | 6 | -42 | 4 | | | 34 | | 2.38 | 304 | 0.12 |
| L Inferior Parietal Lobule | 40 | -34 | -52 | | | 34 | | 2.34 | 216 | 0.12 |
