# Supplementary material for: The Functional Neuroanatomy of Lexical Tone Perception: An Activation Likelihood Estimation Meta-Analysis
Source: Front Neurosci. 2018 Jul 24;12:495. doi: 10.3389/fnins.2018.00495 (PMC6066585; doi:10.3389/fnins.2018.00495)
Supplement: Supplementary file 2 [file Table_2.DOC]

**TABLE S2 | Co-activated regions for tonal tone and other conditions based on FDR-corrected ALE results (FDR-corrected *p* < 0.05,** **minimum cluster = 100 mm^3^).**

| **Brain Region** | **BA** | | **Peak Talairach Coordinates** | | | | **Max. ALE**  **(×10^-2^)** | | **Volume (mm^3^)** |
| --- | --- | --- | --- | --- | --- | --- | --- | --- | --- |
|  |  |  | **x** | **y** | **z** | |  |  |  |
| **Tonal Tone ∩ Non-tonal Tone** |  | | | | | | | | |
| R Superior Temporal Gyrus | 41 | 56 | | -24 | 8 | 1.64 | | 168 | |
| **Tonal tone ∩ Phoneme** | None | | | | | | | | |
| **Tonal Tone ∩ Word Prosody** | None | | | | | | | | |
| **Tonal Tone ∩ Sentence Prosody** | | | | | | | | | |
| L Precentral Gyrus | 9 | | -40 | 4 | 32 | | 1.86 | | 120 |
