# Supplementary material for: The Functional Neuroanatomy of Lexical Tone Perception: An Activation Likelihood Estimation Meta-Analysis
Source: Front Neurosci. 2018 Jul 24;12:495. doi: 10.3389/fnins.2018.00495 (PMC6066585; doi:10.3389/fnins.2018.00495)
Supplement: Supplementary file 3 [file Table_3.DOC]

**TABLE S3 | Brain regions revealed by contrasting tonal tone with other conditions based on FDR-corrected ALE results (FDR-corrected *p* < 0.05, minimum cluster = 100 mm^3^).**

| **Brain Region** | **BA** | **Peak Talairach Coordinates** | | | **Z Score** | **Volume (mm^3^)** |
| --- | --- | --- | --- | --- | --- | --- |
|  |  | **x** | **y** | **z** |  |  |
| **Tonal Tone > Non-tonal Tone** | None | | | | | |
| **Non-tonal Tone > Tonal Tone** | |  |  |  |  |  |
| R Superior Temporal Gyrus | 42 | 58 | -29 | 12 | 2.46 | 360 |
| **Tonal tone > Phoneme** | |  |  |  |  |  |
| R Superior Temporal Gyrus | 41 | 56 | -26 | 8 | 2.2 | 424 |
| **Phoneme > Tonal Tone** | None | | | | | |
| **Tonal Tone > Word Prosody** | None | | | | | |
| **Word Prosody > Tonal Tone** | None | | | | | |
| **Tonal Tone > Sentence Prosody** | |  |  |  |  |  |
| R Superior Temporal Gyrus | 22 | 59 | -21 | 7 | 3.72 | 856 |
| **Sentence Prosody > Tonal Tone** | |  |  |  |  |  |
| R Inferior Frontal Gyrus | 9 | 46 | 12 | 20 | 3.89 | 1104 |
| R Superior Temporal Gyrus | 41 | 42 | -34 | 2 | 2.47 | 560 |
| R Inferior Parietal Lobule | 40 | 36 | -50 | 42 | 2.21 | 552 |
