# Supplementary figures and images for: The Functional Neuroanatomy of Lexical Tone Perception: An Activation Likelihood Estimation Meta-Analysis
Source: Front Neurosci. 2018 Jul 24;12:495. doi: 10.3389/fnins.2018.00495 (PMC6066585; doi:10.3389/fnins.2018.00495)

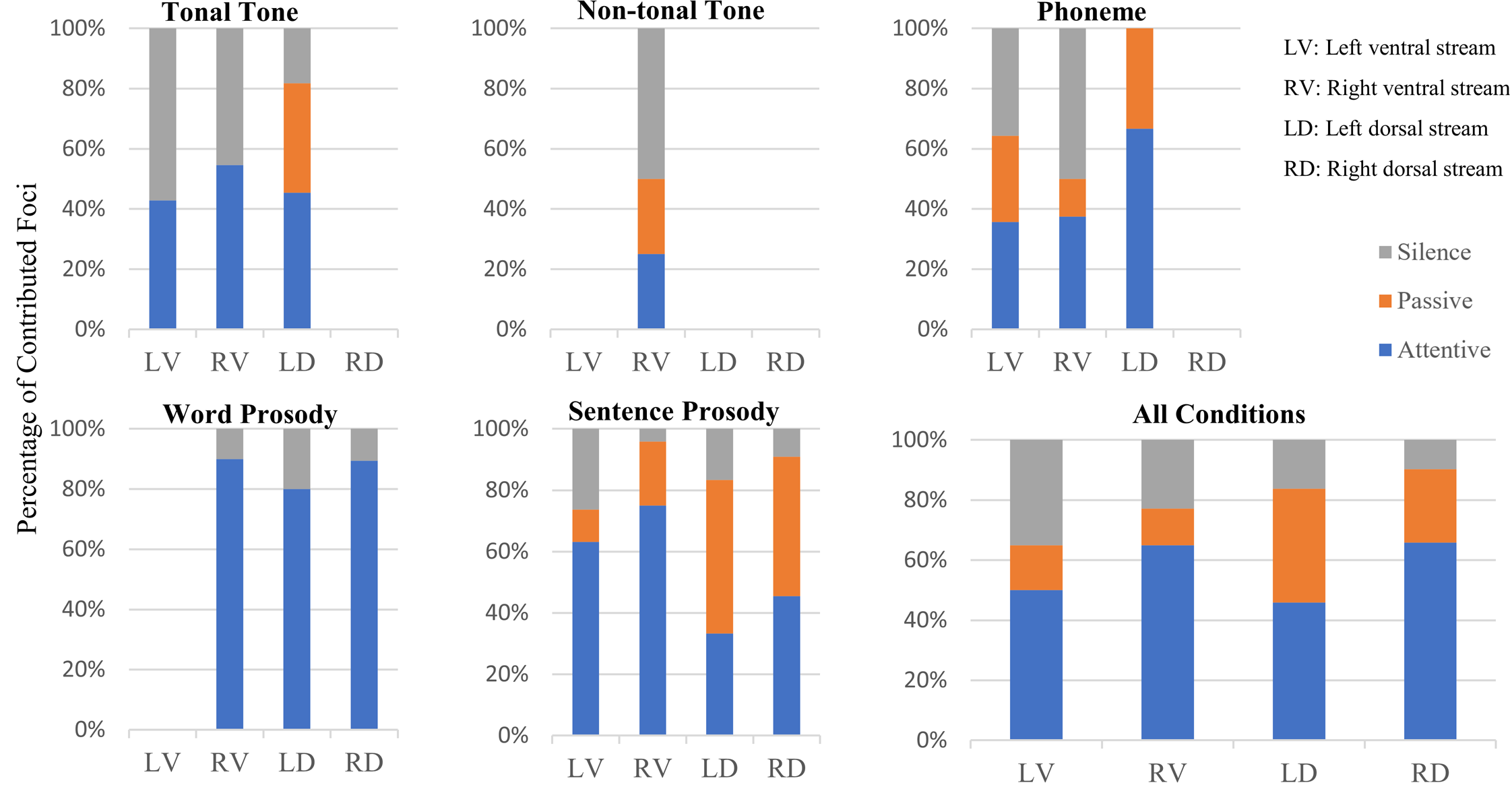

Supplement: Supplementary file 4 [file Image_1.TIF]

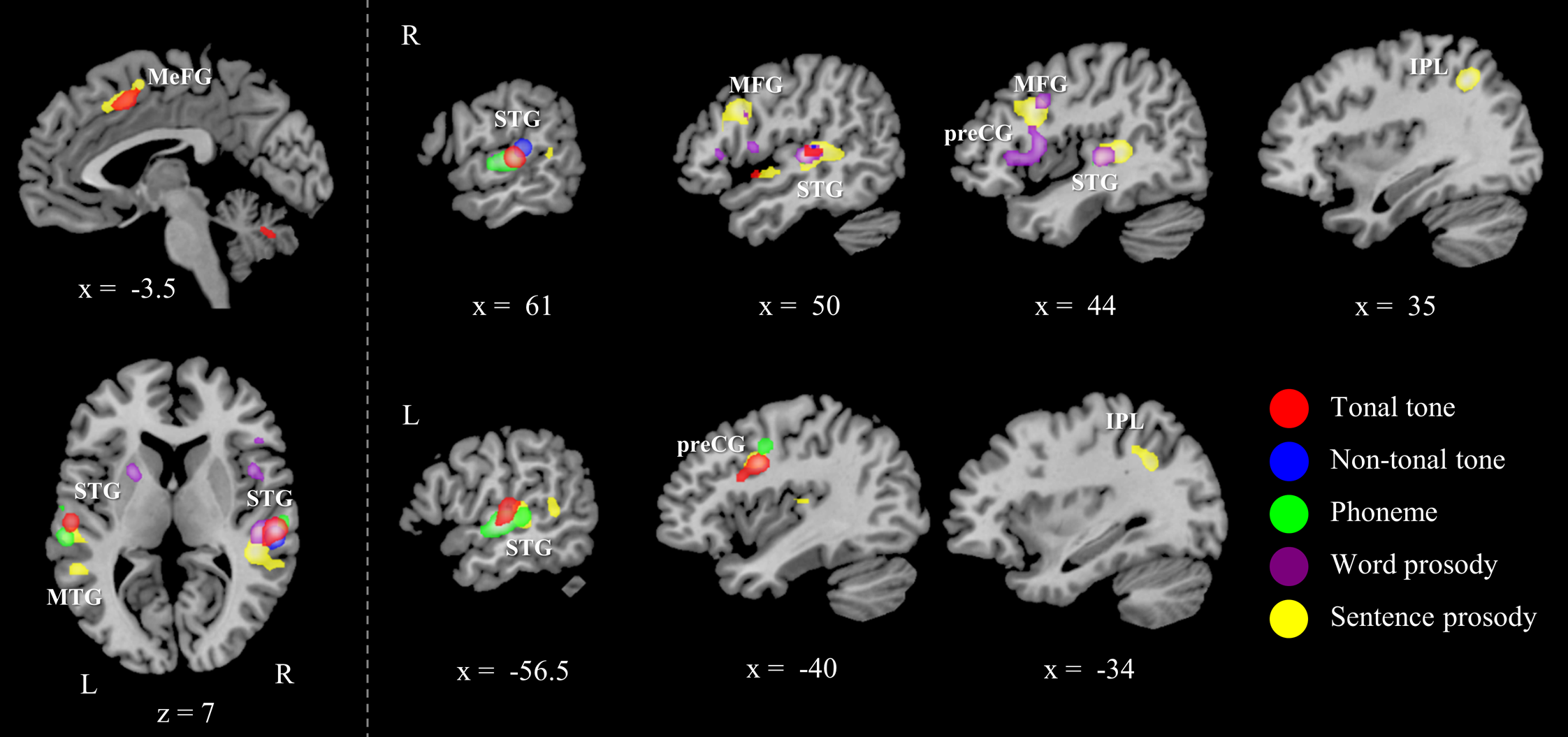

Supplement: Supplementary file 5 [file Image_2.TIF]

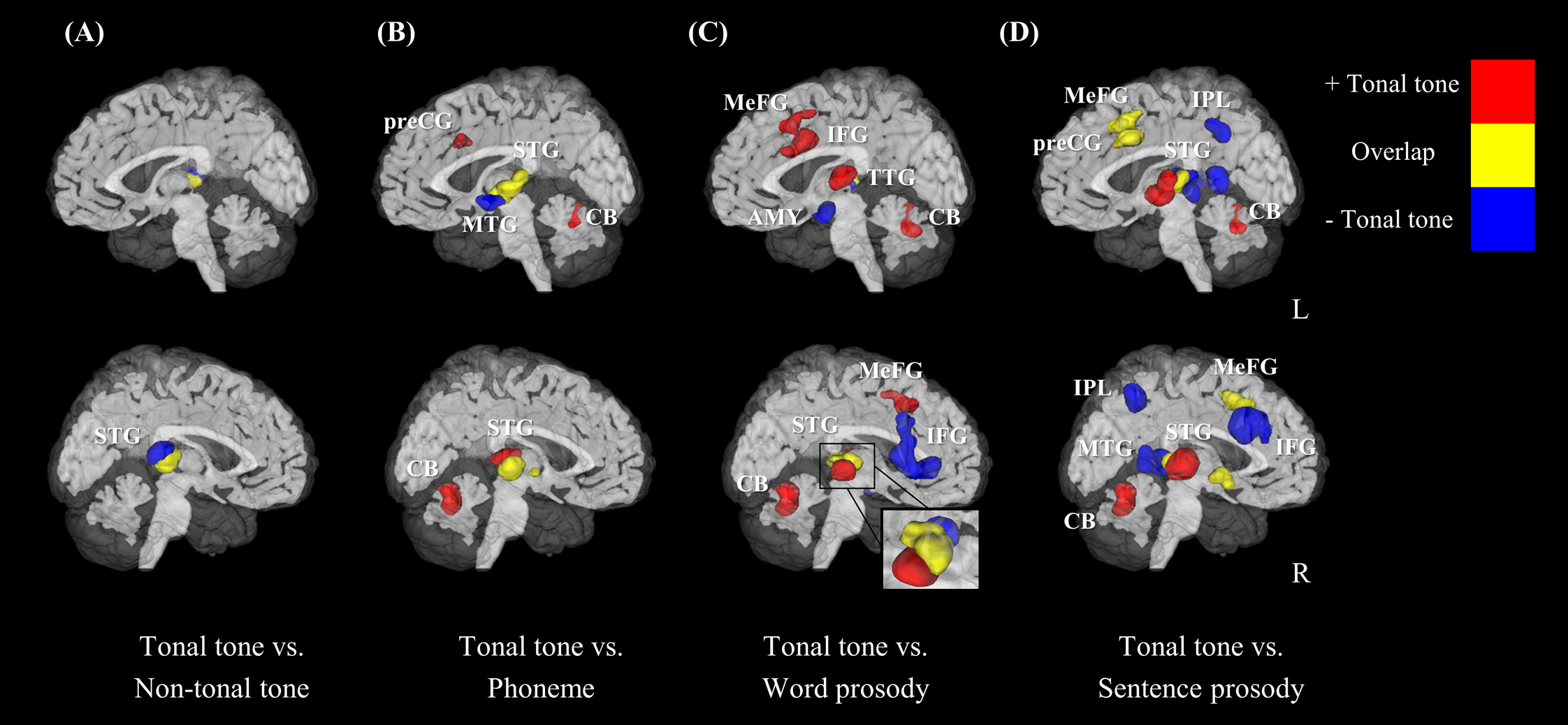

Supplement: Supplementary file 6 [file Image_3.TIF]

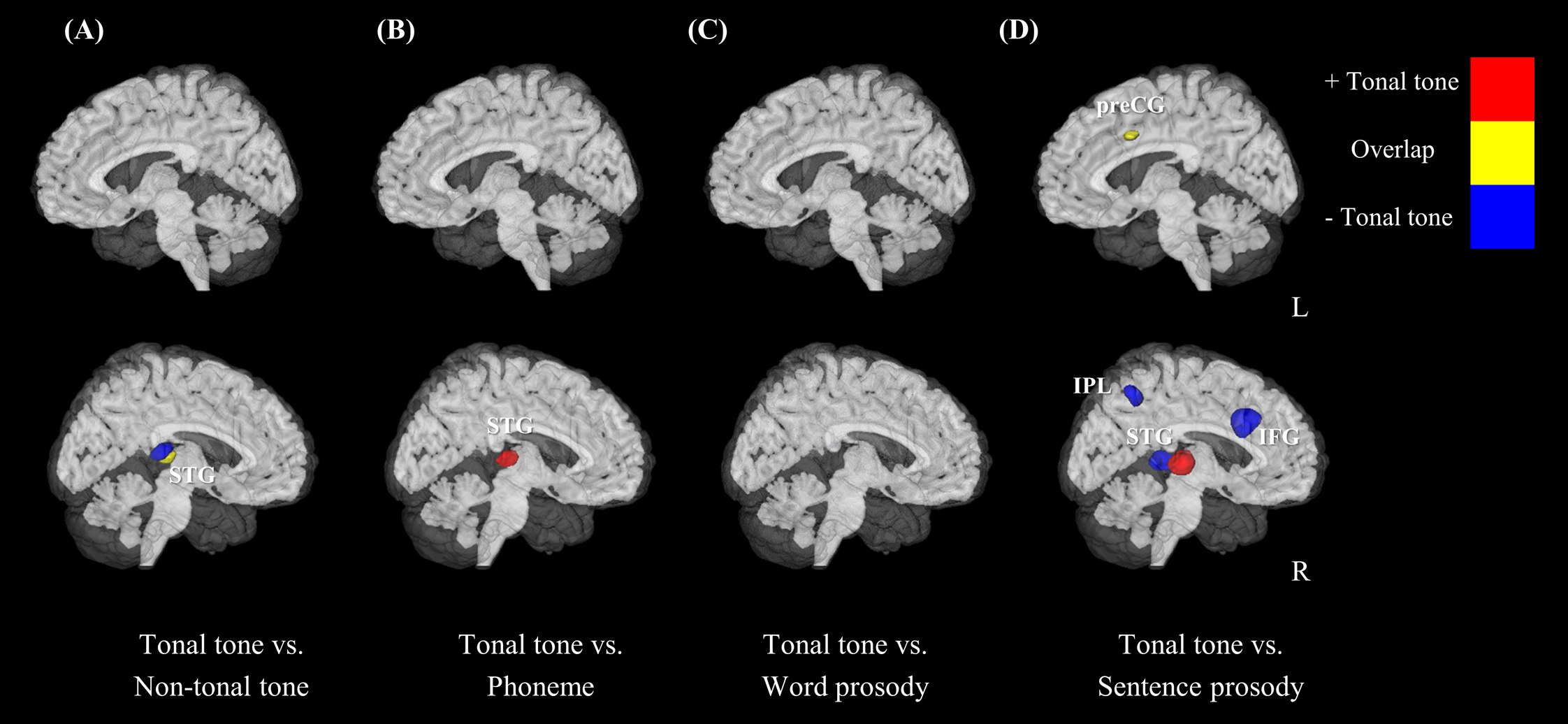

Supplement: Supplementary file 7 [file Image_4.TIF]
